# Butyrate regulates neutrophil homeostasis and impairs early antimicrobial activity in the lung

**DOI:** 10.1016/j.mucimm.2023.05.005

**Published:** 2023-08

**Authors:** Anh Thu Dang, Christina Begka, Céline Pattaroni, Laura R. Caley, R. Andres Floto, Daniel G. Peckham, Benjamin J. Marsland

**Affiliations:** 1 Department of Immunology and Pathology, Central Clinical School, Monash University, Melbourne, Australia; 2 Department of Respiratory Medicine, Leeds Teaching Hospitals NHS Trust, Leeds, United Kingdom; 3 University of Cambridge, Molecular Immunity Unit, Department of Medicine, Cambridge, United Kingdom; 4 Royal Papworth Hospital, Cambridge Centre for Lung Infection, Cambridge, United Kingdom; 5 Leeds Institute of Medical Research, University of Leeds, Leeds, United Kingdom

## Abstract

Short-chain fatty acids (SCFAs) are metabolites that are produced after microbial fermentation of dietary fiber and impact cell metabolism and anti-inflammatory pathways both locally in the gut and systemically. In preclinical models, administration of SCFAs, such as butyrate, ameliorates a range of inflammatory disease models including allergic airway inflammation, atopic dermatitis, and influenza infection. Here we report the effect of butyrate on a bacteria-induced acute neutrophil-driven immune response in the airways. Butyrate impacted discrete aspects of hematopoiesis in the bone marrow resulting in the accumulation of immature neutrophils. During *Pseudomonas aeruginosa* infection, butyrate treatment led to the enhanced mobilization of neutrophils to the lungs as a result of increased CXCL2 expression by lung macrophages. Despite this increase in granulocyte numbers and their enhanced phagocytic capacity, neutrophils failed to control early bacterial growth. Butyrate reduced the expression of nicotinamide adenine dinucleotide phosphate, oxidase complex components required for reactive oxygen species production, and reduced secondary granule enzymes, culminating in impaired bactericidal activity. These data reveal that SCFAs tune neutrophil maturation and effector function in the bone marrow under homeostatic conditions, potentially to mitigate against excessive granulocyte-driven immunopathology, but their consequently restricted bactericidal capacity impairs early control of *Pseudomonas* infection.

## INTRODUCTION

Recent advances have shed light on the contribution of dietary fiber and gut-derived short-chain fatty acids (SCFAs) on host immune homeostasis and immunity. The effects of SCFAs such as propionate, acetate, and butyrate are tremendously pleiotropic and their influence reaches beyond the gastrointestinal tract to peripheral tissues, such as the lung and skin. SCFAs can directly affect immune cells through G-protein coupled receptor engagement, their histone deacetylase (HDAC) inhibitory function, or through diffusion across the cell membrane, not only to maintain homeostasis but also dampen various chronic inflammatory states^1^, ^2^. Neutrophils are the most abundant circulating leukocyte population and are constantly replenished from the bone marrow (BM) during homeostasis. In the context of inflammatory insults, neutrophils are rapidly generated *de novo* and deployed to the circulation in what is termed ‘emergency granulopoiesis’^3^. However, excessive and prolonged mobilization of these effector cells can cause collateral tissue damage.

These short-lived and terminally differentiated granulocytes are the first to arrive at the site of inflammation and are believed to have limited transcriptional activity^4^. Nevertheless, SCFAs have been shown to directly influence neutrophils at the transcriptional level by regulating the production of inflammatory cytokines such as tumor necrosis factor (TNF), possibly through HDAC inhibition^5^, and chemotaxis^6^, ^7^. We have previously shown that gut-derived SCFAs indirectly dampen neutrophil-driven immunopathology during influenza infection via the development of anti-inflammatory Ly6c- monocytes from monocyte-dendritic cell progenitors (MDPs). The protection was mediated through reduced production of CXCL1 by macrophages, thus limiting the infiltration of neutrophils into the lungs and preventing collateral tissue damage. However, the direct effects of SCFAs on neutrophil effector function were not addressed in the study^8^.

Here, we report that butyrate reduces neutrophil bactericidal activity during an acute pulmonary *Pseudomonas aeruginosa* infection, despite the increased mobilization of neutrophils to the lungs and enhanced phagocytic capacity. Butyrate had an intrinsic effect on neutrophil maturation in the BM under homeostatic conditions. Butyrate specifically reduced the bacterial killing capacity of neutrophils *in vivo* and *ex vivo* by decreasing oxygen-dependent reactive oxygen species (ROS) production, through down-regulating the expression of nicotinamide adenine dinucleotide phosphate (NADPH) enzyme complex components. It also limited the expression of secondary granule enzymes, among which lactoferrin has a dual role as an antimicrobial peptide and a transcription factor (TF) during neutrophil development. Taken together, these data advance our understanding of the influence of bacterial metabolites on hematopoiesis, which ultimately tunes peripheral immune responses.

## RESULTS

### Butyrate influences CXCL2 expression and early *Pseudomonas aeruginosa* clearance

We have previously shown an indirect anti-inflammatory effect of SCFAs on neutrophils during pulmonary influenza infection by limiting their mobilization through reduced macrophage-derived production of CXCL1^8^. However, neutrophils also express a receptor for SCFAs, GPR43, that has been reported to influence their chemotaxis^9^, and the extent to which SCFAs directly affect neutrophils remains to be determined. To address this, we used *P. aeruginosa* as an infection model for acute pneumonia, where the early immune response is dominated by neutrophils^10^. Prior to infection, mice were fed a diet with low fiber content for 4 weeks followed by supplementation of their drinking water with the SCFA, butyrate, for at least 2 weeks. Thereafter, adult mice were inoculated with 1 × 10^6^ colony-forming units (CFU) of *P. aeruginosa* strain PAO1 through intratracheal administration, which induced an acute infection with rapid neutrophil-mediated bacterial clearance and no sepsis or death^10^. Cellular infiltration into the lung and bacterial clearance were assessed 18 hours post-infection.

**Fig. 1:**
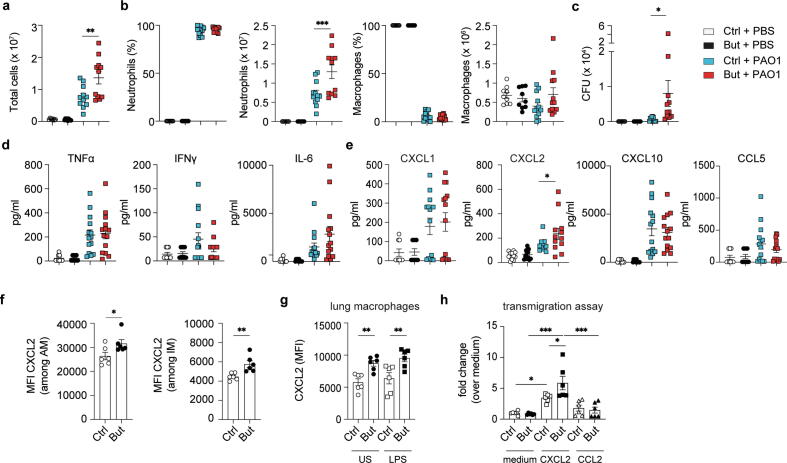
Effect of butyrate on acute *Pseudomonas aeruginosa* immunity. (A) Quantification of the total number of cells in BALF 18 hours post-inoculation with 1 × 10^6^ CFU of *P. aeruginosa* strain PAO1 in Ctrl and But mice. (B) Frequency and quantification of cell types in cytospins of BALF from control and butyrate-treated mice 18 hours after PAO1 infection; (C) Quantification of *P. aeruginosa* CFU in BALF 18 hours post-challenge. (D, E) Cytokine and chemokine production in BALF 18 hours following infection. (F) MFI of CXCR2 on lung AM and IM from Ctrl and But mice. (G) MFI of CXCR2 on Ctrl and But lung macrophages US or stimulated for 2 hours with LPS *in vitro*. (H) transmigration of Ctrl and But neutrophils toward a CXCL2 or CCL2 chemokine gradient after 3 hours. Results are a mean of two independent experiments. Values are expressed as mean ± standard error of mean; *n* = 6–14. Statistical significance was determined with one-way analysis of variance in (A–E, G, H) and Student’s *t* test (unpaired, two-tailed) in (F). * *p* ≤ 0.05, ** *p* ≤ 0.01, *** *p* ≤ 0.001. AM = alveolar macrophages; BALF = bronchoalveolar lavage fluid; But = butyrate-treated; CFU = colony-forming units; Ctrl = control; IFN = interferon; IL = interleukin; IM = interstitial macrophages; LPS = lipopolysaccharide; MFI = mean fluorescence intensity; PAO1 = *P. aeruginosa* strain 1; TNF = tumor necrosis factor; US = unstimulated.

Butyrate did not alter bronchoalveolar lavage fluid (BALF) cellularity in uninfected mice compared to controls, but it enhanced cell infiltration in response to *P. aeruginosa* (Fig. 1A). The number of macrophages was unaltered between control and infected groups, with the increased total cell number in the butyrate-treated animals being explained by enhanced neutrophil recruitment (Fig. 1B). Neutrophils are critical for host defense against Gram-negative bacteria such as *P. aeruginosa* as they are highly cytotoxic and the first innate immune cell type to be recruited to control infection. Despite increased neutrophil mobilization (Fig. 1B), butyrate mice were less efficient in clearing the bacterial infection from the lungs (Fig. 1C), suggesting a change in neutrophil function due to extrinsic or intrinsic effects.

We then assessed the expression of cytokines including tumor necrosis factor α (TNFα), interleukin 6 (IL-6), and interferon γ (IFNγ), which are secreted by pulmonary epithelial cells following TLR4 (LPS) and TLR5 (flagellin) stimulation to activate and recruit immune cells to the site of infection^10^, ^11^. These pro-inflammatory cytokines are detected in BALF as early as 3 hours following microbial challenge^12^. In addition, SCFAs have been reported to directly act on neutrophils and inhibit TNFα and nitric oxide (NO) *in vitro*^5^. We found that the levels of TNFα, IL-6, and IFNγ were comparable in control and butyrate-treated mice 18 hours post-infection, suggesting that butyrate did not dampen pro-inflammatory cytokine production in the airways during infection (Fig. 1D). Alveolar macrophages also secrete IL-1β that is sensed by epithelial cells which in turn produce neutrophil chemoattractants, such as keratinocyte chemoattractant, also known as CXCL1, and macrophage inflammatory protein 2 (MIP-2), also referred to as CXCL2, to further amplify the recruitment of neutrophils^11^. While the levels of CXCL1 and other chemokines including CXCL10 and CCL5 were unaltered, CXCL2 was significantly increased in BALF of butyrate-treated mice (Fig. 1E), suggestive of a CXCL2-mediated neutrophil chemoattraction.

As CXCL2 can be released by TLR4-expressing macrophages in response to LPS^13^, we assessed the production of this chemokine in lung macrophages. We observed a higher baseline CXCL2 expression in butyrate-treated alveolar macrophages (AMs) and interstitial macrophages (Fig. 1F). Although *in vitro* stimulation with LPS did not further increase the level of this cytokine, the increased expression of CXCL2 expression in butyrate-treated lung macrophages was maintained (Fig. 1G). To assess whether neutrophils of butyrate-treated mice displayed enhanced reactivity to CXCL2, we performed a transwell migration assay with bone marrow-derived neutrophils toward a chemokine gradient. While CCL2 did not promote chemotaxis, CXCL2 induced a significantly higher migration of neutrophils from butyrate-treated mice compared to controls (Fig. 1H). Neutrophil chemotaxis was not driven by the SCFA receptors Gpr43 and Gpr109a as their expression was unchanged (Supplementary Fig. 1A).

Collectively, butyrate treatment led to exaggerated airway cellularity during *P. aeruginosa* infection linked to an increased CXCL2 expression by lung macrophages that promotes neutrophil chemotaxis into the lungs.

### Butyrate directly affects neutrophil maturation in the BM during homeostasis

The BM is a primary immune site of hematopoietic stem cell maintenance and harbors an immune cell reservoir ready to be deployed into circulation. Signals from gut microbes, such as bacterial components (TLR ligands), not only maintain myelopoiesis but also play an important role in the control of systemic bacterial infections, including *Listeria monocytogenes*^14^. We have previously shown that bacterial metabolites such as SCFAs can also influence BM hematopoiesis during inflammation of the respiratory tract. Depending on the inflammatory context, SCFAs promote distinct aspects of hematopoiesis. In the context of house dust mite (HDM)-induced allergic inflammation, SCFAs promote proliferation of MDPs and common dendritic cell progenitors (CDPs)^15^; in the context of influenza infection, it enhances the development of MDPs, which restrict exaggerated immunopathology^8^.

**Fig. 2:**
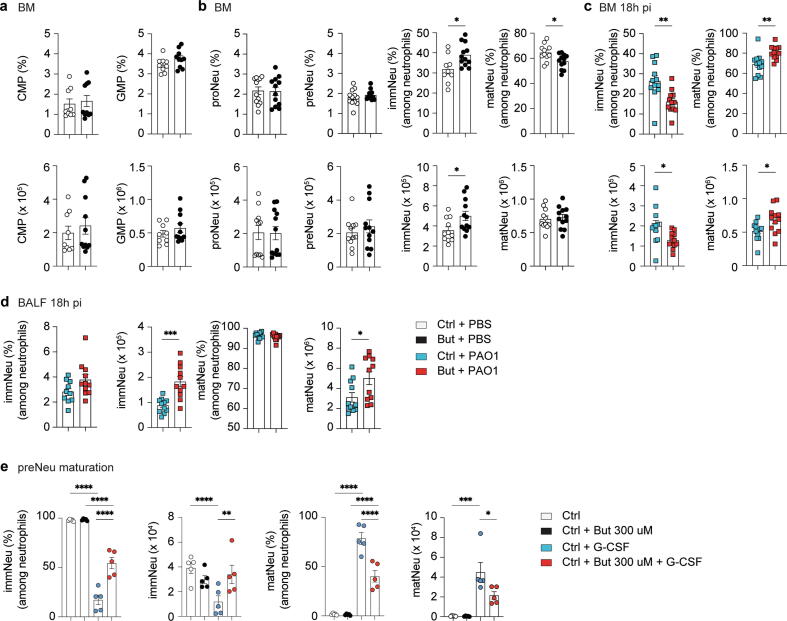
Butyrate affects neutrophil maturation in the BM during homeostasis. (A) Relative representation and quantification of CMP and GMPs in BM of Ctrl and But mice. (B) Frequency, and quantification of proNeu, preNeu, immNeu, and matNeu in BM of Ctrl and But mice. Relative representation and quantification of immNeu and matNeu (C) in BM 18 hours pi and (D) in BALF 18 hours pi. Results are representative of a mean of two independent experiments; E, maturation of FACS-sorted control preNeus cultured for 18 hours with 300 uM butyrate and G-CSF into immNeu and matNeu. Results are expressed as mean ± standard error of mean; *n* = 9–12 per group in (A–D) and n = *5* per group in (E). Statistical significance was determined with Student’s *t* test (unpaired, two-tailed) in (A–D) and One-way analysis of variance in (E). * *p* ≤ 0.05, ** *p* ≤ 0.01, *** *p* ≤ 0.001, **** *p* ≤ 0.0001. BALF = bronchoalveolar lavage fluid; BM = bone marrow; But = butyrate-treated; CMP = common myeloid progenitor; Ctrl = control; G-CSF = granulocyte colony-stimulating factor; GMPs = granulocyte monocyte progenitors; immNeu = immature neutrophils; matNeu = mature neutrophils; PAO1 = *P. aeruginosa* strain 1; PBS = phosphate buffered saline; pi = post-infection; preNeu = preNeutrophils; proNeu = proNeutrophils.

Given the increased *P. aeruginosa* infection-induced neutrophil mobilization into the lungs (Fig. 1B), we examined if butyrate affected proliferation and differentiation potential of granulocyte monocyte progenitors (GMPs), the direct neutrophil precursors, and its precursor the common myeloid progenitors (CMPs)^16^. Under homeostatic conditions, the proportion and total cell numbers of CMPs and GMPs were not altered (Fig. 2A). Butyrate did not affect the early stage of neutrophil maturation when GMPs progress to a highly proliferative pool of cells called ‘pre-neutrophils’ (proNeu, preNeu) (Fig. 2B, Supplementary Fig. 1b)^17^. However, we found that butyrate interfered with the differentiation of neutrophil-committed cells into non-proliferating immature and mature neutrophils (Fig. 2B)^18^. Butyrate treatment maintained a higher immature and lower mature neutrophil proportion and higher immature neutrophil cell number in the BM under homeostasis (Fig. 2B), suggesting a selective effect on terminal neutrophil maturation. Acute microbial insult can elicit targeted expansion and mobilization of myeloid progenitor cells, referred to as emergency granulopoiesis^3^. 18 hours post *P. aeruginosa* challenge, butyrate did not promote myelopoiesis (CMPs) and granulopoiesis (GMPs) (data not shown). However, the homeostatic set point of neutrophil maturation in the BM resulted in rapid deployment of immature neutrophils in butyrate mice in response to *P. aeruginosa* challenge, resulting in a decreased immature neutrophil proportion and cell number (Fig. 2C). Moreover, the homeostatic set point of neutrophil maturation in the BM was evident in the airways, as we observed an increased infiltration of neutrophils of both maturation states into the lungs (Fig. 2D).

To assess if butyrate had a direct effect on terminal neutrophil maturation, we FACS-sorted the neutrophil precursors (preNeu) of control mice and assessed their proliferative capacity *in vitro*. PreNeus predominantly matured into neutrophils with an immature phenotype in the absence of stimulation and in the presence of butyrate (Fig. 2E). In the presence of granulocyte colony-stimulating factor (G-CSF) the neutrophil-committed precursor cells developed mainly into mature neutrophils. However, the differentiation of G-CSF-stimulated control preNeus was modulated toward a higher immature and lower mature neutrophil proportion and cell number in the presence of butyrate (Fig. 2E), similar to our observation *in vivo* (Fig. 2B).

Taken together, butyrate did not promote steady-state myelopoiesis and granulopoiesis but specifically altered late-stage neutrophil maturation reflected by an increased immature neutrophil pool in the BM. Despite this shift in terminal neutrophil maturation in homeostasis, both granulocyte stages accumulated and were recruited to the lung in response to bacterial infection. Overall, butyrate treatment resulted in an enhanced immature neutrophil recruitment to the airways as compared to controls, reflective of the BM environment at steady-state.

### Butyrate affects the transcriptional landscape of BM immature and mature neutrophils under homeostatic conditions

Given the shift in neutrophil maturation under homeostatic conditions, we FACS-sorted immature and mature neutrophils from control and butyrate BMs based on their CXCR2 expression. Immature neutrophils are CXCR2-, while mature neutrophils upregulate this chemokine receptor, which also mediates chemotaxis during inflammation^19^, ^20^.

**Fig. 3:**
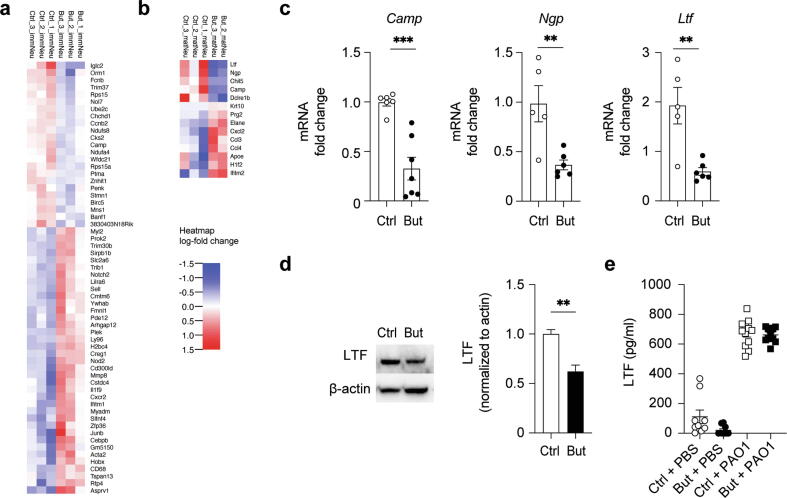
Butyrate alters neutrophil transcriptional landscape under homeostatic conditions. Differential gene expression heat-map of FACS-sorted (A) immature and (B) mature neutrophils from Ctrl and But mice under homeostasis; C, gene expression (fold change) of *Camp*, *Ngp,* and *Ltf* from Ctrl and But total neutrophils; D, immunoblot of uninfected *ex vivo* total neutrophils and quantification of LTF band normalized to β-actin loading Ctrl; E, cytokine expression of LTF in bronchoalveolar lavage fluid of Ctrl and But mice 18 hours post-infection. Results are a mean of two independent experiments for (C, E) or are representative of data from two independent experiments (D). Values are expressed as mean ± standard error of mean; *n* = 5–7 per group in (C, E). For immunoblotting *n* = 3–4 mice per group were pooled. Statistical significance was determined with Student’s *t* test (unpaired, two-tailed) in (C, D) and with one-way analysis of variance in (E). ** *p* ≤ 0.01, *** *p* ≤ 0.001. But = butyrate-treated; *Camp* = cathelicidin; Ctrl = control; *Ltf* = lactoferrin; mRNA = messenger RNA; *Ngp* = neutrophil granule protein; PAO1 = *P. aeruginosa* strain 1; PBS = phosphate buffered saline.

RNA sequencing was performed, and principal component analysis revealed a distinct signature between BM neutrophils from control and butyrate-treated mice (data not shown). Differential gene expression analysis (Fig. 3A) and subsequent pathway analysis (Reactome) showed downregulated pathways involving neutrophil degranulation (*Camp*, *Creg1*, *Orm1*, *CD68*, *Cxcr2*, *Sell*, *Cmtm6*, *Mmp8*, *Sirpb1b*) and transcriptional regulation of granulopoiesis (*Cebpb*, *H2bc4*) in butyrate immature neutrophils compared to controls. Gene expression analysis on mature BM neutrophils revealed a small set of differentially expressed genes between control and butyrate-treated mice under steady-state conditions (FDR0.1) (Fig. 3B), as these are unstimulated cells and terminally differentiated with limited transcriptional activity^4^. Nevertheless, key pathways affected were interleukin signaling (*Ccl3*, *Ccl4*, *Cxcl2*), antimicrobial peptides, and neutrophil degranulation (*Camp*, *Elane*, *Ltf*, *Prg2*). We confirmed by reverse transcription-polymerase chain reaction (PCR) that total neutrophils from butyrate-treated animals expressed significantly lower transcript levels of cathelicidin (*Camp*), lactoferrin (*Ltf*), but also of other secondary neutrophil granule enzymes such as neutrophil granule protein (*Ngp*) (Fig. 3C). In addition, immunoblotting revealed a lower LTF protein level in butyrate-treated total neutrophils (Fig. 3D). Interestingly, lactoferrin is not only a secondary granule protein but also a TF active in early neutrophil development^21^. Despite the decreased amount of intracellular lactoferrin, degranulation of this pre-formed secondary granule was not affected in neutrophils from butyrate-treated mice. The level of extracellular lactoferrin in BALF from control and butyrate-treated mice was comparable, suggesting there is a decreased availability of this enzyme when taking into account the increased neutrophil cell number in the lung (Fig. 1B).

Together, these data suggest that butyrate modulates discrete aspects of the neutrophil transcriptional landscape under homeostatic conditions in the BM. Thereby, neutrophil responses in the periphery could be tuned by specifically altering the production and availability of enzymes that are pre-stored in secondary granules during maturation.

### Butyrate reduces bactericidal activity but enhances phagocytosis

**Fig. 4:**
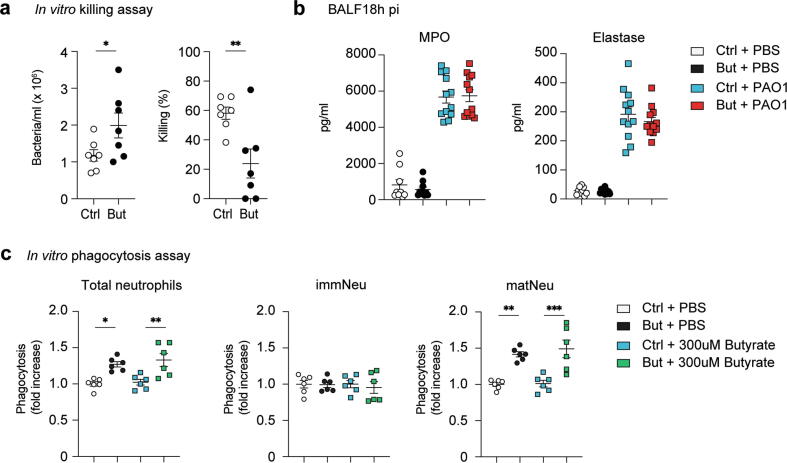
Butyrate reduces bacterial clearance capacity but enhances phagocytosis. (A) Quantification of *Pseudomonas aeruginosa* after co-culture with Ctrl or butyrate neutrophils for 2 hours to assess for *in vitro* killing capacity. (B) Myeloperoxidase and elastase expression in BALF of uninfected or *P. aeruginosa*- challenged Ctrl and But mice. (C) Phagocytosis of PE-labeled immunoglobulin G beads by total, immature and mature bone marrow neutrophils after 2 hours incubation expressed as fold change over control neutrophils. Results are a mean of two independent experiments. Values are expressed as mean ± standard error of mean, *n* = 6–12 per group. Statistical analysis was determined with Student’s *t* test (unpaired, two-tailed) in (A) and One-way analysis of variance in (B, C). * *p* ≤ 0.05, ** *p* ≤ 0.01, *** *p* ≤ 0.001. BALF = bronchoalveolar lavage fluid; But = butyrate-treated; Ctrl = control; immNeu = immature neutrophils; matNeu = mature neutrophils; mRNA = messenger RNA; PAO1 = *P. aeruginosa* strain 1; PE = phycoerythrin; PBS = phosphate buffered saline.

The RNA sequencing of steady-state immature and mature neutrophils suggested degranulation, and hence effector function, was dysregulated in neutrophils from butyrate-treated animals (Figs. 3A and 3B). Despite an enhanced neutrophil mobilization to the BALF and lungs following *P. aeruginosa* infection (Fig. 1A), the bacterial load remained higher in butyrate-treated mice compared to controls (Fig. 1C), suggesting inhibition of effector functions. To investigate this, we performed an *in vitro* killing assay by co-culturing *ex vivo* neutrophils isolated from control and butyrate-treated mice with *P. aeruginosa* and assessed their bactericidal capacity. *Ex vivo* neutrophils from butyrate-treated mice had a lower killing capacity compared to controls as shown by the higher number of surviving bacteria (Fig. 4A). Neutrophils use diverse effector functions for eliminating pathogens including phagocytosis or rapid release of azurophilic granules containing myeloperoxidase (MPO) and elastase into the extracellular space or phagolysosome^4^, ^22^. No difference in MPO and elastase degranulation was observed in BALF (Fig. 4B) despite higher neutrophil infiltration in butyrate mice compared to controls (Fig. 1C). Thus, it cannot be excluded that neutrophils from butyrate-treated animals degranulate less azurophilic enzymes into the extracellular space on a per cell basis (Fig. 4B). Clearance of pathogens by both phagocytosis and subsequent intracellular killing is another key function of neutrophils. SCFAs were reported to decrease neutrophil phagocytic capacity resulting in impairment of *A. actinomycetemcomitans* containment^23^. In contrast, we found that *ex vivo* BM neutrophils from butyrate-treated mice were more phagocytic compared to controls measured by the engulfment of fluorescently labeled immunoglobulin (Ig)G beads. The increased phagocytosis was specific to mature neutrophils and unchanged in immature phagocytes (Fig. 4C).

Overall, *in vivo* exposure to butyrate ‘primed’ BM neutrophils with an enhanced phagocytic capacity. Nevertheless, effective elimination of intracellular pathogens requires further antimicrobial mechanisms following phagocytosis. These data indicated that other effector mechanisms were affected accounting for the reduction in bacteria clearance *in vitro* and *in vivo*.

### Butyrate impairs ROS-mediated bacterial clearance in neutrophils

**Fig. 5:**
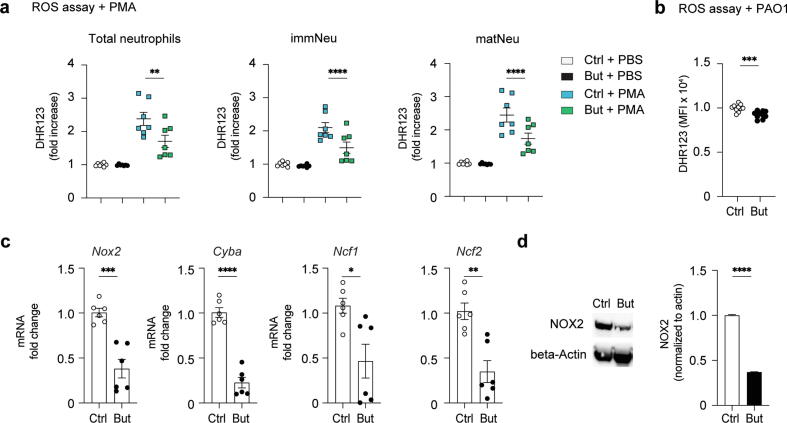
Butyrate impairs ROS-mediated bacterial clearance in neutrophils. A, fold increase of MFI expression of DHR 123 in total, immature and mature BM neutrophils of Ctrl or But mice as a ROS measurement following treatment with PBS or 20 nM PMA for 45 minutes; B, MFI of DHR123 in Ctrl and butyrate BM neutrophils following co-incubation with *Pseudomonas aeruginosa* for 2 hours; C, gene expression of *Nox2*, *Cyba*, *Ncf1*, and *Ncf4* of BM neutrophils; D, western blot of uninfected *ex vivo* neutrophils and quantification of NOX2 band normalized to loading control. Results are a mean of two independent experiments (A–C) or are representative of data from two independent experiments (D). Values are expressed as mean ± standard error of mean, *n* = 7–10 per group in (A, B), *n* = 6 per group in (C). For immunoblotting *n* = 3–4 mice per group were pooled. Statistical analysis was determined with one-way analysis of variance in (A) and Student’s *t* test (unpaired, two-tailed) in (B–D). * *p* ≤ 0.05, ** *p* ≤ 0.01, *** *p* ≤ 0.001, **** *p* ≤ 0.0001. BM = bone marrow; But = butyrate-treated; Ctrl = control; DHR = dihydrorhodamine; immNeu = immature neutrophils; matNeu = mature neutrophils; MFI = mean fluorescence intensity; mRNA = messenger RNA; ROS = reactive oxygen species; PAO1 = *P. aeruginosa* strain 1; PE = phycoerythrin; PMA = Phorbol 12-Myristate 13-Actetate; PBS = phosphate buffered saline.

Following phagocytosis, generation of ROS in the phagolysosome by the NADPH oxidase 2 (NOX2) is a potent mechanism to control and eliminate intracellular pathogens. Butyrate was shown to enhance NADPH activity and ROS production to increase anti-bacterial activity in macrophages^21^. To assess if butyrate affected respiratory burst in neutrophils, we measured cellular ROS production in *ex vivo* BM neutrophils using Dihydrorhodamine 123 (DHR123), which is a non-fluorescent ROS indicator that emits green fluorescence on oxidation. ROS content in unstimulated neutrophils of control and butyrate-treated mice were comparable (Fig. 5A). However, following *ex vivo* Phorbol 12-Myristate 13-Actetate (PMA) stimulation, butyrate-treated neutrophils produced less intracellular ROS than control neutrophils. The reduced ROS production was not restricted to mature neutrophils but was also observed in immature cells (Fig. 5A). Similarly, on co-culturing of purified BM neutrophils with *P. aeruginosa*, neutrophils of butyrate-treated exhibited a reduced oxidative burst compared to controls (Fig. 5B).

The NADPH oxidase is a multi-protein complex consisting of the main catalytic domain NOX2 and CYBA, another phagosome membrane subunit, and other cytosolic components (NCF1, NCF2, NCF4). Following activation, the NADPH complex is recruited and assembled at the phagosome membrane to generate superoxide anion radicals (O2^.−^) that are converted into highly bactericidal hydrogen peroxide (H_2_O_2_) in the phagosome lumen. We found that gene expression of *Nox2* and also of *Cyba*, *Ncf1,* and *Ncf4* was significantly reduced in unstimulated neutrophils of butyrate-treated mice compared to controls (Fig. 5C). Moreover, immunoblotting showed that NOX2 protein levels were also reduced in neutrophils of butyrate mice under homeostatic conditions (Fig. 5D).

Taken together, the microbicidal defect of butyrate neutrophils was a result of a decreased intrinsic oxidative burst capacity due to reduced expression of oxygen-dependent NADPH oxidase complex components required for cellular ROS conversion following activation.

### Discussion

We have previously reported the anti-inflammatory effect of SCFAs on allergic and viral pulmonary inflammation involving the gut–bone marrow–lung axis. The BM, which is home to a diverse array of progenitor cells, emerged as a central site that is modulated by SCFAs to shape peripheral immune responses. The impact of SCFAs on BM hematopoiesis, specifically myelopoiesis, is highly context-dependent; SCFAs induced patrolling monocytes during influenza^8^ and less mature DCs during HDM-induced asthma^15^ to dampen pulmonary inflammation.

Here we have identified a role for butyrate in establishing the set point of neutrophil responsiveness during homeostasis and inflammation by modulating terminal differentiation of neutrophils and by altering their transcriptional landscape. We found that butyrate treatment resulted in neutrophils with a decreased capacity for NADPH-dependent ROS production, possibly to prevent immunopathological damages, which however was at the expense of bactericidal function *in vivo* and *in vitro*.

In prior studies, the SCFA-driven phenotypical changes to progenitor cells only became evident during inflammation, which is likely due to as yet unexplored molecular signatures that set those cells on their functional trajectories. Aligned with prior observations, we did not detect any changes in BM hematopoiesis and myelopoiesis under homeostatic conditions in butyrate-treated animals (data not shown). However, butyrate-treated mice did exhibit preferential accumulation of immature neutrophils as compared to mature neutrophils in the BM at steady-state as compared to controls. Increasing evidence supports a linear development of proliferative preNeutrophils (preNeu) to immature to fully functional mature neutrophils that are dictated by a highly coordinated expression of TFs accompanied by functional changes^17^. Hence, distinct sets of TFs were reported to be active in different developmental stages in neutrophils^24^. Certain early neutrophil C/EBP TFs have been associated with specific granule expression such as C/EBPα^25^ with primary granule enzymes such as MPO, and C/EBPε and C/EBPδ with secondary granules such as LTF^26^. Primary granules are mainly pre-formed at the GMP stage, while secondary granules are expressed at the preNeu and immature neutrophil stage^17^. Our RNA sequencing and immunoblotting results revealed a decreased lactoferrin expression in unstimulated neutrophils from butyrate-treated animals, suggesting that butyrate interferes with the generation of these pre-formed granules around the immature developmental phase and that this alteration is maintained in the mature stage. Moreover, the gene expression of other secondary (specific) granules such as Camp and Ngp were also affected by butyrate.

Lactoferrin (Ltf) is an iron-binding glycoprotein found in secondary granules that functions in defense against microbial infections through sequestration of iron and thus depriving bacteria of nutrients essential for growth^27^. However, lactoferrin has a dual role as a well-recognized TF as it harbors a nuclear localization signal^28^. Its gene expression is particularly prominent in the early neutrophil developmental stage^24^. The decreased lactoferrin expression in butyrate mice could explain the maturation shift between immature and mature neutrophils in our model, suggesting that a certain level of lactoferrin needs to be sustained during the early developmental stages for full neutrophil maturation. This finding also implies that peripheral signals such as bacterial metabolites could instruct steady-state neutrophil development in the BM and dictate their cell fate, function, and peripheral responses. Neutrophils can be subjected to reprogramming as has been observed in an experimental mouse model of KRAS-driven lung adenocarcinoma to favor tumor growth^29^. Contrastingly, these granulocytes were shown to adopt an immune-suppressive phenotype (N2 neutrophils) to reduce brain injury in the presence of a peroxisome proliferator-activated receptor-γ (PPARγ) agonist^30^. Nevertheless, those butyrate neutrophils which were able to reach full maturation, despite lower Ltf expression, did not possess the full range of neutrophil effector function and showed ‘defects’ in respiratory burst, possibly attenuating neutrophil activation. Similarly, lactoferrin-deficient mice display normal early neutrophil development, migration, phagocytosis, granule release, and antimicrobial response but have an impaired respiratory burst in mature neutrophils in response to treatment with PMA^31^. Here, we found that butyrate controlled ROS production by down-regulating the expression of the main catalytic domain *Nox2* but also of the other NADPH complex components *Cyba*, *Ncf1*, and *Ncf4*. Similarly, butyrate has been linked to reduction in murine arthritis development through decreasing NADPH oxidase subunit p22phox (Cyba) expression and thus, oxidative stress in endothelial cells^32^. A previous study suggested that *in vitro* pre-treatment with butyrate reduced p47phox (Ncf1) phosphorylation in rat neutrophils^33^. However, this is in contrast to findings by Schulthess et al. who found that butyrate-treated macrophages produced more intracellular ROS after PMA stimulation or *Salmonella* infection^21^. Nevertheless, these inconsistent findings could be due to differences in protocols, such as SCFA concentration and cell activation. In response to the reduced bactericidal activity, enhancement of neutrophil mobilization and phagocytic capacity could be a compensatory mechanism to control the infection, which requires further investigation.

Taken together, neutrophils are critical effector cells of the innate immune system providing immediate host defense against microbial challenges using a combination of oxidative and non-oxidative effector systems. Here, we found that butyrate modulated the oxidative arm by inhibiting NADPH-dependent ROS production prior to inflammation. Moreover, butyrate partially regulated the non-oxidative defense mechanism by targeting the expression of secondary (specific) granule enzymes, such as lactoferrin, that also act as a transcriptional activator during neutrophil development. As the sequential expression of certain TFs is highly regulated and accompanied by an ordered upregulation of effector functions during different neutrophil developmental stages^24^, the influence of microbial components or their by-products during early development in the BM can have lasting effects on the arsenal of effector functions in fully mature neutrophils. Our findings suggest that despite the short-lived nature of neutrophils, changing the transcriptomic and functional signatures of these granulocytes in the BM could have relevant implications in chronic diseases such as cystic fibrosis (CF) where recurrent *P. aeruginosa* infections cause neutrophil-mediated immunopathology, associated with accelerated lung function decline^34^, ^35^. Neutrophil dysregulation, as a result of heterogeneity or activation status within the neutrophil population, is implicated in pathogenic conditions such as CF and systemic lupus erythematosus (SLE)^18^, ^34^, ^36^. By tuning down excessive neutrophil responses in these chronic and pre-disposed conditions, SCFAs might function to establish a “healthy” set point of the immune system where infections can be controlled in the absence of exaggerated immunopathology.

The aforementioned effect on neutrophils, ranging from maturation to activity, was not specific to butyrate but was partially recapitulated with acetate and propionate. Contrary to butyrate, these two SCFAs did not alter the proportion of immature and mature neutrophils in the BM. However, they enhanced neutrophil phagocytic capacity and reduced ROS production similar to butyrate (Supplementary Figs. 2A–C).

While dietary SCFA supplementation could have therapeutic potential in viral respiratory infections and allergic airway inflammation^8^, ^15^, further investigation is required to determine its potential impact on bacteria-driven or neutrophilic lung disorders.

## MATERIAL AND METHODS

### Mice

Four weeks-old C57BL/6 female mice were purchased from WEHI (Melbourne, Australia) and housed under specific pathogen-free facilities at Monash University, Alfred Research Alliance, Melbourne, Australia. Mice were fed a low-fiber diet (Speciality Feeds diet SF09-28) for 4 weeks prior to SCFA supplementation and throughout the experiment. Mice received sodium butyrate (Sigma-Aldrich, St. Louis MO), sodium acetate (Sigma-Aldrich, St. Louis MO), or sodium propionate(Sigma-Aldrich, St. Louis MO) in the drinking water at a final concentration of 300 mM for 2 weeks prior to *P. aeruginosa* infection and throughout the experiment. Diet was purchased from Speciality Feeds, Australia. All animal experiments were approved and performed in accordance with animal protection guidelines of the Alfred Research Alliance Animal Ethics Committee, Melbourne, Australia.

### *P. aeruginosa* infection

*P. aeruginosa* strain PAO1 (ATCBAA47, ATCC) was cultured in tryptic soy broth (TSB, BD Biosciences) to an OD600 of 0.5. Bacteria were pelleted, washed twice with phosphate-buffered saline (PBS), and resuspended in PBS. The bacterial inoculum was estimated based on OD600 and verified by plating serial dilutions on agar plate to determine CFU. 10-12 week-old mice were anesthetized with a mixture of ketamine (100 mg/kg) and xylazine (10 mg/kg) and infected intratracheally with 1 × 10^6^ CFU in 30 μl of sterile PBS. Mice were sacrificed 18 hours post-infection and bronchoalveolar lavage (BAL) was performed. Total CFU was enumerated by serial dilution and plating on TSA agar. Total CFU was converted to CFU/ml of BALF.

### Flow cytometry

Phenotyping and characterization of various cell types in BM and lungs were performed by flow cytometry. Lung mouse tissue was digested using collagenase IV (BioConcept, Worthington, Lakewood, NY) in Dulbecco’s Modified Eagle Media (DMEM) for 45 minutes at 37°C. Samples were then filtered through a 70-μm cell strainer and washed in DMEM 10% FCS. BM cells were isolated by flushing mouse femurs with PBS 10% FCS using a 25-gauge needle and syringe and filtered through a 70-μm cell strainer. Cells were counted and then stained for flow cytometry. To differentiate progenitor and neutrophil populations in the BM, cells were stained with antibodies to lineage cocktail, CD115 (BD Biosciences, clone 2B8), SiglecF (BD Biosciences, clone 2B8), CD117 BUV117 (BD Biosciences, clone 2B8), Sca1 APCFire (Biolegend, clone D7), FcgR biot (Miltenyi, clone 93), CD34 BV421 (Biolegend, clone SA376A4), CD11b BV510 (BD Biosciences, clone M1/70), Ly6G Pecy5 (eBioscience, clone 1A8-Ly6g), CXCR2 Pecy7 (Miltenyi, clone REA942) and streptavidin BUV395 (BD Biosciences). Neutrophils in lungs and BALF were identified as using antibodies to CD45-biot (Miltenyi, clone 30-F11), CD11c APCCy7 (Biolegend, clone N418), SigF AF647 (BD Biosciences, clone E50), CD11b PB (Biolegend, clone M1/70), Ly6G Pecy5 (eBioscience, clone 1A8-Ly6g), CXCR2 Pecy7 (Miltenyi, clone REA942) and streptavidin BV786 (BD Biosciences). For intracellular staining, cells were fixed with 4% paraformaldehyde (PFA) followed by permeabilization with 0.1% saponin. Cells were stained with antibodies to CXCL2 (Invitrogen, clone 40605) followed by anti-rat IgG2b FITC (Biolegend, clone RTK4530). All cells were acquired on a Fortessa (BD Biosciences) and analysis was performed using FlowJo software (Tree Star, Ashland, OR).

### RNA sequencing

BM cells were harvested by flushing femurs with DMEM 10% FCS and immature (lin^−^ CD117^lo/int^ Sca1^−^ Ly6G^+^ CD11b^+^ CXCR2^−^) and mature (lin^−^ CD117^lo/int^ Sca1^−^ Ly6G^+^ CD11b^+^ CXCR2^+^) neutrophils were stained as described and sorted by flow cytometry. Immediately after sorting, cells were pelleted by centrifugation and RNA was isolated using the Quick-RNA MiniPrep kit (Zymo Research) according to manufacturer. RNA quantity was measured with Qubit (Invitrogen) and RNA quality was assessed on the Fragment Analyzer. For library preparation, total RNA was first amplified using the Clontech Smart-seq v4 ultra low kit before preparation with the NEBNext Ultra RNA Library Prep Kit for Illumina. Resulting libraries were sequenced on an Illumina NovaSeq platform (PE150) at a depth of 20M reads per sample. Raw fastq files were processed using RNASik v1.5.0^37^ with default settings. Briefly, alignment was performed using STAR v2.5.2b against the *Mus musculus* GRCm38 reference genome with the associated gene-model GTF. featureCounts^38^ was used to quantify read counts before import into Degust.

### *In vitro P. aeruginosa* killing assay

BM was flushed with RPMI 10% FCS 1% Pen/Strep and neutrophils were negatively isolated according to the manufacturer (Miltenyi). 5 × 10^5^ neutrophils were infected at a multiplicity of infection of 10 with *P. aeruginosa* in HBSS 10% FCS. Neutrophils were incubated with bacteria in tubes with end-over-end rotation (6rpm) for 120 minutes at 37°C. Bacterial CFU was enumerated via serial dilution with H_2_0 pH11 and growth on agar plates at 37°C. The percentage of killed *P. aeruginosa* was calculated by dividing the number of CFU that grows in the presence of neutrophils by the number of CFU that grows in the absence of neutrophil.

### Oxidative Burst Assay (ROS assay)

1 × 10^6^ BM cells were incubated with 1 ug/ml Dihydrorhodamine 123 (DHR) (ThermoFisher) in RPMI and stimulated with 20 nM PMA (Sigma-Aldrich) and 300 uM sodium butyrate (Sigma-Aldrich) for 45 minutes at 37°C. Cells were washed with PBS, stained for flow cytometry and the fluorescence intensity of neutrophil subsets was measured.

### Phagocytosis assay

1 × 10^6^ BM cells were incubated with IgG-PE beads (Cayman Chemical) at a 1:100 dilution for 2 hours at 37°C. After incubation, cells were washed with PBS, stained for flow cytometry and analyzed.

### Transwell assay

BM was flushed with RPMI 10% FCS 1% Pen/Strep and neutrophils were negatively isolated according to the manufacturer (Miltenyi). Cells were cultured o/n in RPMI 10% FCS 1% Pen/Strep 25 ng/ml GM-CSF (PeproTech) at 37°C 5% CO_2_. 5 × 10^5^ neutrophils were seeded into the 3 um pore 12-well insert. Medium containing CXCL2 or CCL2 was added to the bottom well. After 3 hours incubation at 37°C 5% CO_2_ transmigrated cells were counted.

### *In vitro* lung macrophage assay

Lung mouse tissue was digested using collagenase IV (BioConcept, Worthington, Lakewood, NY) in DMEM for 45 minutes at 37°C with gentle agitation. Samples were then filtered through a 70-μm cell strainer and washed in DMEM 10% FCS. Cells were resuspended in DMEM 10% FCS 1% P/S and culture in non-tissue treated plates o/n at 37°C 5% CO_2_. The following day, the supernatant was removed and macrophages were detached from the wells with 5 mM EDTA. 1 × 10^5^ macrophages were plated in 96-well plate and stimulated with 20 ng/ml LPS for 2 hours at 37°C 5% CO_2_. After incubation, cells were washed with PBS, stained for flow cytometry, and analyzed.

### Neutrophil precursor assay

Neutrophil progenitors (preNeus) of control mice were FACS-sorted from BM cells. 3 × 10^4^ preNeus were seeded into 96-well plate and stimulated with 300 uM Butyrate (Sigma) and 10 ng/ml G-CSF (Peprotech) for 16 hours at 37°C 5% CO_2_. After incubation, cells were washed with PBS, stained for flow cytometry, and analyzed for maturation into immature and mature neutrophils.

### ELISA

TNFa, IFNg, IL-6 (Thermo Fisher Scientific), and CXCL2 (R&D) protein levels were measured by enzyme-linked immunosorbent assay (ELISA). Using a LEGENDplex panel CXCL1, CXCL10, and CCL5 (BioLegend) proteins in BALF were quantified by flow cytometry. Neutrophilic activity in BALF of mice 18 hours post-infection was quantified using myeloperoxidase (R&D), elastase (R&D), and lactoferrin ELISA kit (St John's Laboratory) according to the manufacturer’s instruction.

### Immunoblot

Neutrophils were purified from BM using MACS (130-097-658, Miltenyi) according to the manufacturer and were lysed, and protein extract was quantified by BCA (Thermo Fisher Scientific). Total cell lysates were run on SDS-PAGE (Thermo Fisher Scientific), followed by transfer on methylcellulose membranes (Thermo Fisher Scientific). Proteins were detected using primary antibodies against lactoferrin (PA5-95513, Thermo Fisher Scientific), NOX2 (ab129068, Abcam), β-actin (4967S, Cell Signaling), and HRP-conjugated secondary antibodies.

### Quantitative PCR

RNA was isolated from MACS-purified neutrophils using TRIzol (Life Technologies). Complementary DNA synthesis was performed using FIREScript complementary DNA Reverse Transcriptase (Solis BioDyne). Quantitative PCR was performed using PowerUp SYBR Green (Life Technologies) and run on the QuantStudio 6 machine (Life Technologies). Relative mRNA expression of *Ltf*, *Camp*, *Ngp*, *Ccl3*, *Ccl4*, *Nox2*, *Cyba*, *Ncf1*, *Ncf2*, *Ncf4*, *Gapdh,* and *Polr2a* were assessed by quantitative RT-PCR using the following primer sets: *Ltf* forward 5’-CCGCTCAGTTGTGTCAAGAA-3’ and reverse 5’-AGACTTCAGCTGCCACAGGT-3’; *Camp* forward 5’-CGAGCTGTGGATGACTTCAA-3’ and reverse 5’-CTCCTTCACTCGGAACCTCA-3’; *Ngp* forward 5’-CCACTCCGCCTTCTAGTCAG-3’ and reverse 5’-TCCAGGAAGTCGCAGTCTTT-3’; *Nox2* forward 5’-CCCTTTGGTACAGCCAGTGAAGAT-3’ and reverse 5’-CAATCCCGGCTCCCACTAACATCA-3’; *Cyba* forward 5’-CCCTCCACTTCCTGTTGTC-3’ and reverse 5’-CCCTCACTCGGCTTCTTT-3’; *Ncf1* forward 5’-GATGAAGACAAAGCGAGGTT-3’ and reverse 5’-CAGATACATGGATGGGAAATAG-3’; *Ncf4* forward 5’-GCTTCACCAGCCACTTTGTT-3’ and reverse 5’-AGGGCTGTTCTTGCTCTCTG-3’; *Gapdh* forward 5’-CGTCTTCACCACCATGGAGA-3’ and reverse 5’-CGGCCATCACGCCACAGTTT-3’; *Polr2a* forward 5’-GAGTCCAGAACGAGTGCATGA-3’ and reverse 5’-ACAGGCAACACTGTGACAATC-3’.

### Statistical analysis

Student’s t test (unpaired, two-tailed) or one-way analysis of variance test was used to calculate significance levels between treatment groups with GraphPad Prism 9. Graphs and figure legends are annotated with the level of significance between the test groups. A *p*-value < 0.05 was considered significant.

## AUTHOR CONTRIBUTIONS

A.D. and B.J.M. designed the study. A.D. and C.B. performed experiments. A.D., C.B., and C.P. analyzed data. A.D., C.B., C.P., L.C., R.F, D.P., and B.J.M. provided supervision, critical analysis, and discussions. A.D. and B.J.M. wrote the paper. All authors edited the paper.

## DECLARATIONS OF COMPETING INTEREST

B.J.M. is on the Editorial Board of Mucosal Immunology. No other authors have a conflict of interest.

## FUNDING

This work was supported by the Cystic Fibrosis Trust [SRC 012]; fellowships to B.J.M. from the National Health and Medical Research Council (NHMRC), Australia (Grant number 1154344) and the Victorian Endowment for Science, Knowledge, and Innovation (VESKI).
