# Supplementary material for: Butyrate regulates neutrophil homeostasis and impairs early antimicrobial activity in the lung
Source: Mucosal Immunol. 2023 Aug;16(4):476–85. doi: 10.1016/j.mucimm.2023.05.005 (PMC10412508; doi:10.1016/j.mucimm.2023.05.005)
Supplement: Supplementary data 1 [file mmc1.pdf]

SUPPLEMENTARY DATA

Butyrate regulates neutrophil homeostasis and impairs early antimicrobial activity in the lung.

Anh Thu Dang[1], Christina Begka[1], Céline Pattaroni[1], Laura R. Caley[4], R. Andres Floto[3], Daniel G. Peckham[2, 4], Benjamin J. Marsland[1]

[1] Department of Immunology and Pathology, Central Clinical School, Monash University, Melbourne, Australia
[2] Department of Respiratory Medicine, Leeds Teaching Hospitals NHS Trust, Leeds, United Kingdom.
[3] University of Cambridge, Molecular Immunity Unit, Department of Medicine; Royal Papworth Hospital, Cambridge Centre for Lung Infection
[4] Leeds Institute of Medical Research, University of Leeds, United Kingdom

Corresponding author: benjamin.marsland@monash.edu

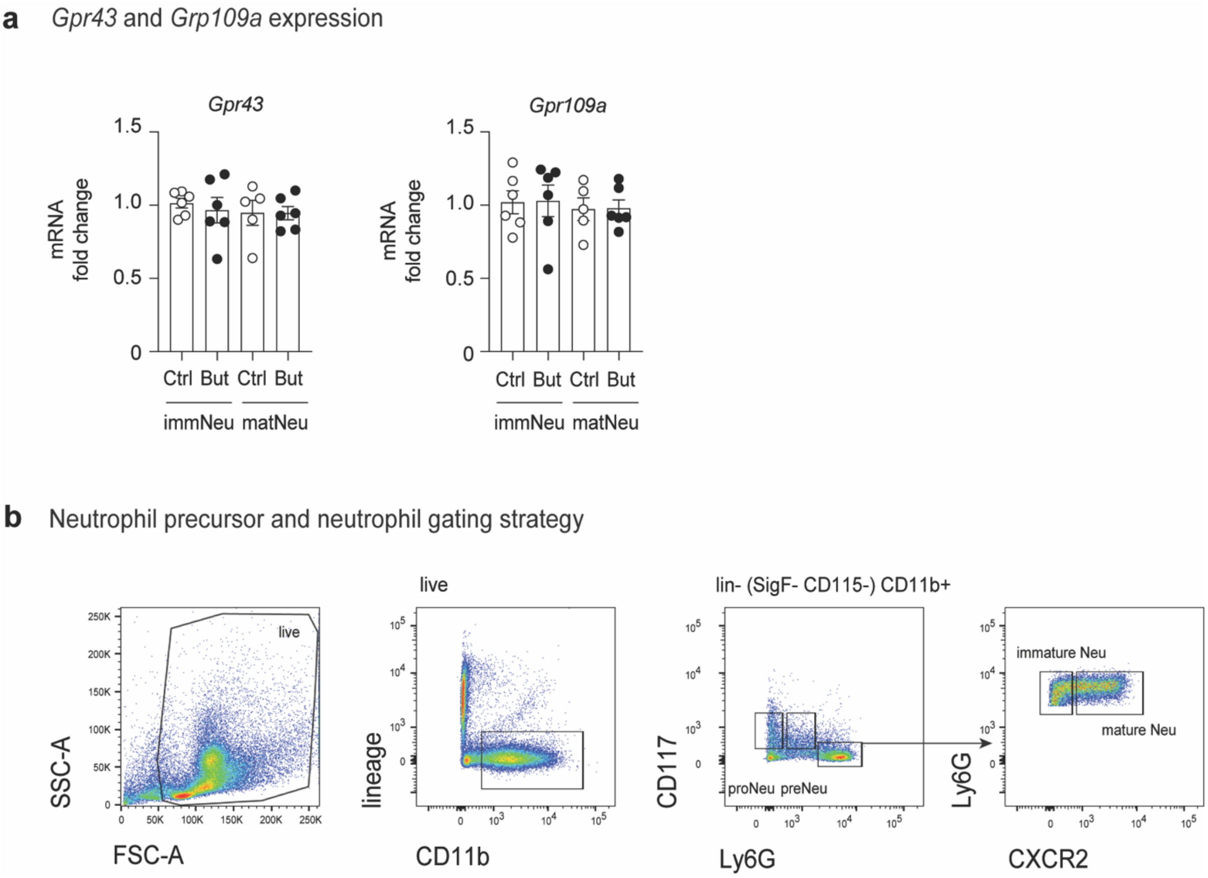

Supplementary Figure 1. Neutrophil precursor and neutrophil gating strategy in the bone marrow. a Gpr43 and Gpr109a mRNA expression in FACS-sorted immature (immNeu) and mature neutrophils (matNeu). **B** FACS gating strategy for the identification of the neutrophil precursors proNeutrophils (proNeu) and preNeutrophils (preNeu) and immature neutrophils (immNeu) and mature neutrophils (matNeu) in bone marrow. Results are a mean of two independent experiments (a). Values are expressed as mean ± SEM, n = 5-6 per group. Statistical analysis was determined with One-way ANOVA.

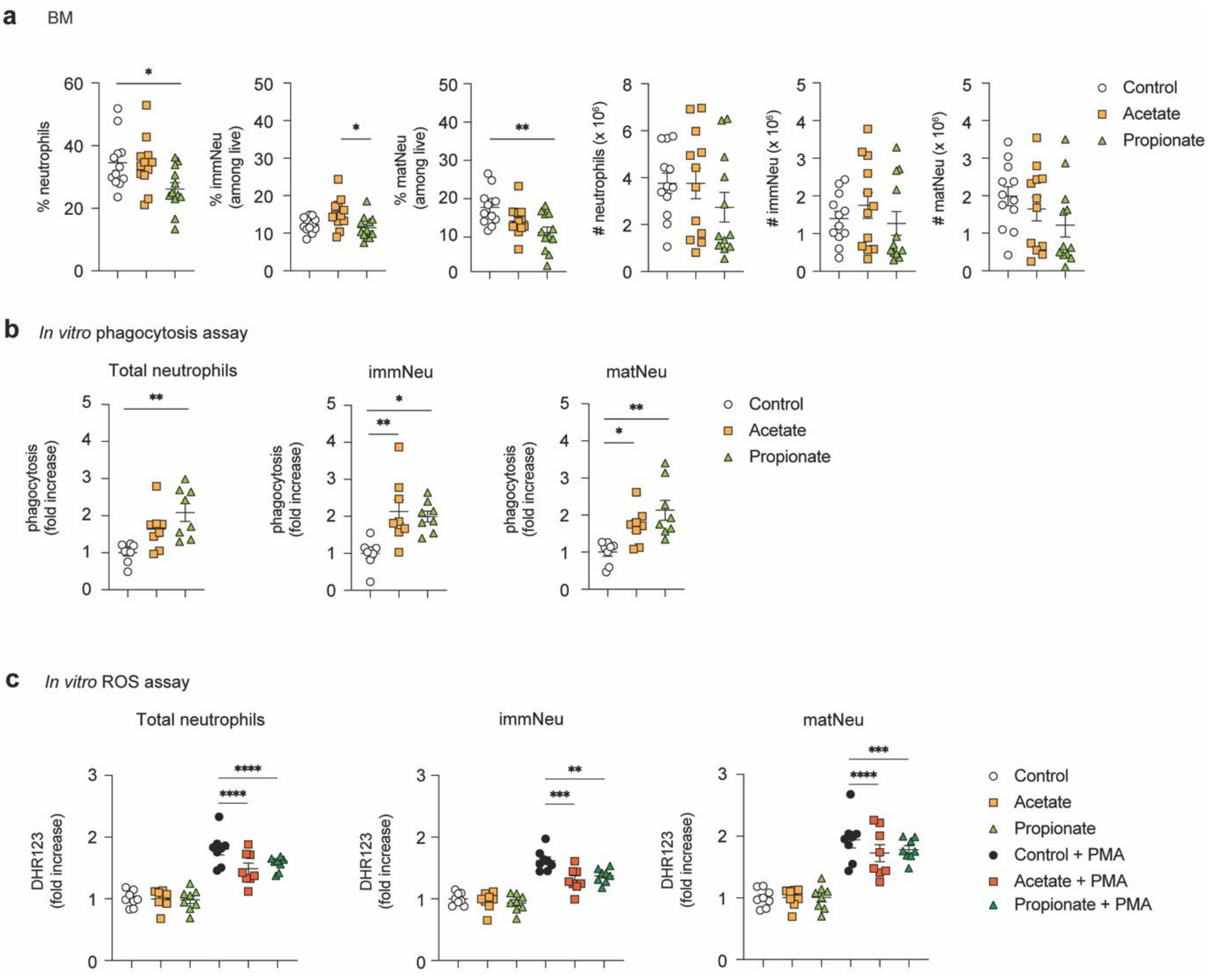

Supplementary Figure 2. Acetate and propionate mimic changes in neutrophil effector function. a Frequency and quantification of total neutrophils, immature neutrophils (immNeu) and mature neutrophils (matNeu) in bone marrow (BM) of control (ctrl), acetate- and propionate-treated mice. **b** Phagocytosis of PE-labelled IgG beads by total, immature and mature BM neutrophils of control, acetate- and propionate-treated mice after 2h incubation expressed as fold change over control neutrophils. **b** Fold increase of mean fluorescence intensity (MFI) expression of dihydrorhodamine (DHR) 123 in total, immature and mature BM neutrophils of control (ctrl), acetate- and propionate-treated mice following treatment with PBS or 20 nM PMA for 45min. Results are a mean of two independent experiments (a-c). Values are expressed as mean ± SEM, n = 8 per group. Statistical analysis was determined with One-way ANOVA. *p $\leq$ 0.05, **p $\leq$ 0.01, ***p $\leq$ 0.001, ****p $\leq$ 0.0001.
